# Propranolol as therapy for cerebral cavernous malformations: a cautionary note

**DOI:** 10.1186/s12967-022-03360-4

**Published:** 2022-04-05

**Authors:** Robert Shenkar, Thomas Moore, Christian Benavides, Rhonda Lightle, Matthew R. Detter, Nicholas Hobson, Romuald Girard, Dorothy DeBiasse, Mary Patrucco, Carol Gallione, Joseph M. Zabramski, Douglas A. Marchuk, Issam A. Awad

**Affiliations:** 1 Department of Neurological Surgery, University of Chicago Medicine and Biological Sciences, 5841 S. Maryland, MC 3026, Chicago, IL 60637 USA; 2 Department of Molecular Genetics and Microbiology, Duke University, Durham, NC USA; 3 Department of Neurosurgery, Barrow Neurological Institute, Phoenix, AZ USA

## Letter to the Editor:

Encouraged by case histories, a clinical trial (Treat_CCM) is investigating the effect of propranolol at doses between 40 and 80 mg/day on patients with cerebral cavernous malformations (CCM) [1]. Oldenburg and coworkers [2] showed that propranolol decreased lesion development and rescued barrier function in CCMs in *Ccm3/(Pdcd10)*^*iECKO*^ model of CCM disease when given propranolol orally at 12.5 mg/kg/day (equivalent to 70 mg/day for a 70 kg human adult [3, 4]) up to age P21 via mother’s milk, and then at 100 mg/kg/day (equivalent to 560 mg/day for a 70 kg human adult [3, 4]) from ages P21 to P28. Our recently published preclinical study showed that oral propranolol at 50 mg/kg/day (equivalent to 280 mg/day for a 70 kg human adult [3, 4]), given at later ages (starting at P21 for 35 or 90 days), significantly decreased lesion burden in two murine models of CCM disease, but did not affect hemorrhage [5]. In the present pre-clinical study, we reduced the mouse dose to 15 mg/kg/day for 35 days to test a human equivalent adult dose of 84 mg/day [3, 4].

The *Pdcd10*^*ECKO*^model (*Pdcd10*^*fl/fl*^*Pdgfb-iCreERT2*), in which 25 mg of tamoxifen was injected at P6 [5], included 72 mice, utilizing 13 mice for hemodynamics and 59 mice (28 males, 31 females) for lesion burden and hemorrhage assessments. Guidelines for objectivity in preclinical research, were followed, including randomization, blinding of outcome assessment, appropriate sample size estimation based on the primary outcome, and prespecified statistical analysis plan, as has been reported previously [5]. Methods to acquire and analyze data for hemodynamics (contractility and heart rate), lesion burden (percent lesion volume per mouse brain volume, primary outcome), acute and chronic hemorrhage, and attrition were described previously [5].

**Fig. 1:**
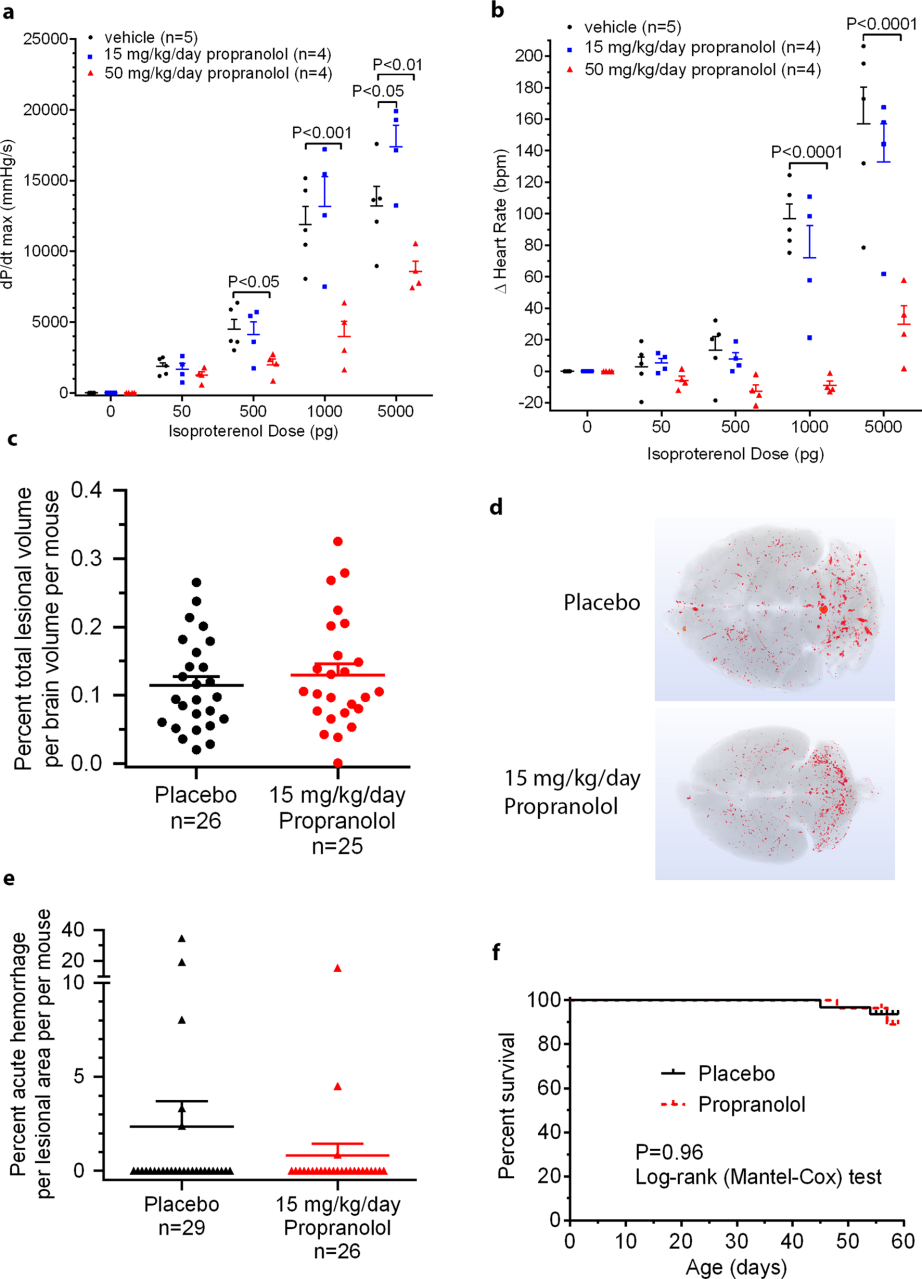
Effect of propranolol in the *Pdcd10*^*ECKO*^ murine model of cerebral cavernous malformation disease. Propranolol treated mice at 50 mg/kg/day showed more of a significant rightward shift in the dose response curve in **a** left ventricular contractility (dP/dt max) and **b** change (Δ) of heart rate in beats per minute (bpm) with increasing concentrations of isoproterenol than those treated at 15 mg/kg/day. Data were analyzed by two-way repeated measures analysis of variance, post hoc independent samples t-test. **c** Propranolol treatment (15 mg/kg) for 35 days, (dose started P21) did not affect lesion burden compared with placebo controls (P = 0.466, t test). Three placebo mice and one propranolol-treated mouse were excluded as outliers per prespecified criteria. Their inclusion did not result in therapeutic effect. **d** Representative micro-computed tomography with average lesion burdens showed no effect of propranolol in *Pdcd10*^*ECKO*^ brains. **e** Propranolol treatment did not affect acute hemorrhage compared to placebo controls (P = 0.527, Mann–Whitney test). **f** Propranolol treatment did not affect attrition compared to placebo controls (P = 0.96, Log-rank Mantel-Cox test). Data are expressed as mean + standard error of the mean in **a**, **b**, **c** and **e**

Mean ± standard error of the mean weights at harvest were similar (P = 0.166, t test) between placebo (17.42 ± 0.30, n = 29) and propranolol-treated (18.11 ± 0.39, n = 26) mice. In contrast to pronounced decreases observed from propranolol at 50 mg/kg/day, propranolol treatment at 15 mg/kg/day had a minimal effect on left ventricular contractility (Fig. 1a) or heart rate (Fig. 1b), when challenged with isoproterenol, and only at highest doses. Propranolol treatment at 15 mg/kg/day did not affect CCM lesion burden (Fig. 1c, d), acute hemorrhage (Fig. 1e), chronic hemorrhage (scant Perls stained non-heme iron in 4 out of 29 placebo and in 2 out of 26 propranolol treated mice) nor attrition (Fig. 1f).

These preclinical results suggest that the human doses used in the Treat_CCM trial may not be effective on lesional development and growth in patients with CCM disease. This letter is a cautionary note about the frequent lack of publication of negative pre-clinical results, and assumptions of dose–effect in clinical trials based on mechanistic studies which often deploy higher doses than those tolerated in humans.

## Data Availability

The datasets used and/or analyzed during the current study are available from the senior authors on reasonable request.

## Abbreviation

CCM: Cerebral cavernous malformation
